# The prevalence of undiagnosed Prediabetes/type 2 diabetes, prehypertension/hypertension and obesity among ethnic groups of adolescents in Western Canada

**DOI:** 10.1186/s12887-020-1924-6

**Published:** 2020-01-23

**Authors:** Shelley Spurr, Jill Bally, Carol Bullin, Diane Allan, Erick McNair

**Affiliations:** 1 Faculty of Nursing, College of Nursing, University of Saskatchewan, 104 Clinic Place, Saskatoon, Saskatchewan S7N 2Z4 Canada; 2 Faculty of Medicine, College of Medicine, University of Saskatchewan, Health Sciences Building, Saskatoon, Saskatchewan S7N 5E5 Canada; 3 Strategic Analyst, College of Nursing, University of Saskatchewan, Saskatoon, Saskatchewan S7N 5E5 Canada

**Keywords:** Adolescents, Prediabetes, Type 2 diabetes, Hypertension, Obesity

## Abstract

**Background:**

An increased incidence of type 2 diabetes in youth is occurring worldwide. While diverse ethnic groups are disproportionately affected by type 2 diabetes, studies that explore ethnic differences and undiagnosed prediabetes/type 2 diabetes in adolescents are scarce. This paper compares the prevalence of undiagnosed prediabetes and type 2 diabetes and the associated risk factors among various ethnic groups of adolescents living in Western Canada.

**Methods:**

The data for this study were derived from two previous studies in which 396 adolescents, aged 14 to 19 years and living in a western Canadian province, were screened for undiagnosed prediabetes, diabetes, and any associated risk factors. Risk was determined by demographics, family history, anthropometric measurements (body mass index, BMI), blood pressure (BP), and HbA1c. Descriptive and inferential statistics (SPSS) were used to establish both risk and prevalence for prediabetes and type 2 diabetes. Chi-square analyses were done to determine if the risk factors occurred at higher frequencies in certain ethnicities.

**Results:**

Based on BP, BMI, and HbA1c measurements, several statistically significant differences were identified in relation to ethnicity. Many of the adolescents had increased HbA1c levels, with 27.3% considered high risk and 2.3% in the prediabetes range; these high risk and prediabetes groups were heavily represented by Filipino (46%), Indigenous (22%), and European (10%) adolescents. Notable prevalence of prehypertension (17.7%) and hypertension (21.7%) were reported in European (59%) followed by Filipino (50%) and Indigenous (26%) adolescents. Higher numbers of adolescents in the European and Filipino ancestry groups had two or more risk factors (BP, BMI, & HbA1c) for developing type 2 diabetes in relation to the adolescents from the Indigenous group.

**Conclusions:**

Ethnic adolescent groups demonstrate a notable prevalence of undiagnosed prediabetes and type 2 diabetes. Specifically, a significant number of Filipino adolescents had both increased HbA1c and blood pressure that has gone undetected. Earlier onset of type 2 diabetes is congruent with an increased risk of developing diabetes-associated complications and, ultimately, diabetes-related morbidity and mortality at a younger age. Future studies should explore how genetic and/or environmental factors among ethnic groups may contribute to early onset hypertension and prediabetes/type 2 diabetes.

## Background

The incidence of type 2 diabetes in youth is increasing worldwide. In Canada, the incidence of type 2 diabetes in children and adolescents has been estimated at 1.54 per 100,000 per year [1]. In the United States of America (USA), the overall incidence has been increasing by 7.1% annually (from 9 cases per 100,000 youths per year in 2002–2003 to 12.5 cases per 100,000 per year in 2011–2012) [2]. Other countries are experiencing similar increases in the numbers of children and youth being diagnosed with type 2 diabetes [3, 4]. The prevalence of this chronic disease is rapidly increasing and is a serious concern within this population and across nations [5].

Canada is rich with diverse ethnicities and is the highest among the G8 countries in terms of immigration, with over one-fifth of the population being foreign-born [6]. The children of some of these ethnic groups (African, Arab, Asian, and Indigenous) are disproportionately affected by type 2 diabetes [7]. This is also the case in the USA, where ethnic groups including Hispanics, Non-Hispanic Blacks (NHB), Asians, and Native Americans have an increased incidence of type 2 diabetes [2, 8, 9]. The large SEARCH for Diabetes in Youth Study investigated the incidence of type 2 diabetes in Asian, Pacific Islander (Filipino), and Asian Pacific Islander populations and found the incidence rate to be 12.1 per 100,000 for youth aged 10–19 years, similar to the general USA rates [10]. A recent study from the United Kingdom reported an increased prevalence in young people of South Asian, African, and African-Caribbean descent [4]. A study conducted in Denmark found that ethnic Turkish, Arab, and Pakistani adolescents were more likely to have a familial history and increased risk of developing type 2 diabetes [11]. Overall, there is clear evidence of the increasing numbers of ethnic youth who are being diagnosed with type 2 diabetes across the globe.

Canada has experienced significant increases in the number of Indigenous youths developing prediabetes and type 2 diabetes. A recent study conducted in Manitoba by Amed et al. [12] reported that 100/227 (44%) of newly diagnosed cases of type 2 diabetes were Indigenous youth. Other Canadian studies have also found that Indigenous children, along with other ethnic youth, are experiencing increased prevalence of diagnosed type 2 diabetes [13, 14].

Although the prevalence of type 2 diabetes among youth of varying ethnicities has been documented, most of the data are from diabetes clinics and are focused on etiology, diagnosis, and clinical features. Findings from our previous investigation into the prevalence of undiagnosed prediabetes and type 2 diabetes indicate a disconcerting prevalence of prediabetes (10%) in adolescents living in three northern and predominantly Indigenous communities in Western Canada [13, 15]. Therefore, a second study [15] was undertaken with adolescents living in a mid-sized Western Canadian city and found that 29.3% were at high risk of developing either diabetes or prediabetes and 2.6% were classified in the prediabetes range. However, no reports to date consider the unique differences between the various adolescent ethnic groups participating in the abovementioned studies in relation to the prevalence of undiagnosed prediabetes and type 2 diabetes and the associated risk factors of adolescents living in this Western Canadian province.

This article reports the demographic and risk factors for prediabetes and type 2 diabetes along with the prevalence of undiagnosed prediabetes and type 2 diabetes in adolescents from diverse ethnicities, including those of Indigenous, Filipino, and European descent. To our knowledge, this is the first report to compare undiagnosed prediabetes among the various ethnic groups of adolescents living in this Western Canadian province.

## Methods

Data for this analysis are derived from two previous studies [13, 15] that were approved by the University Biomedical Ethics board and the northern health regions, and that received written permission from the superintendents of the school districts and the principals of the five participating high schools. A detailed description of the study methods is published elsewhere [13]. Common procedures were employed in the two studies. Before any contact with potential participants, a letter was sent home to inform the adolescents and parents of the purpose and procedures of the study, confidentiality, and their right to withdraw. Then, at each school, the school principal and the Principal Investigator (PI) of the study visited all of the classes to explain the study and invite all students to participate. During these classroom visits, the students were reassured that they were not required to participate and refusal would not jeopardize their course standing. Written parental consent and student assent were obtained from each participant aged ≤17. Adolescents aged ≥18 were granted permission by the ethics board and school districts to provide their own consent. The PI and three Registered Nurses (RNs) conducted the diabetes and risk factor screening in a quiet room within the participating high schools.

### Design

Adolescents who live in Western Canada were screened for prevalence of undiagnosed prediabetes and type 2 diabetes and the associated risk factors. Identification of risk for prediabetes and type 2 diabetes was assessed through the collection of demographic data, family history, anthropometric measurements, blood pressure (BP), and hemoglobin A1c (HbA1c) blood glucose levels.

### The sample

The sample included high school students from three northern and remote Canadian predominantly Indigenous communities (*n* = 143) and from two urban high schools in a mid-sized (~ 250,000 population) western Canadian city (*n* = 253) who were assessed for risk for prediabetes and type 2 diabetes. The response rates for each school are reported in Table 1. No participants withdrew from the study. Qualifying criteria included adolescents who were 14–19 years old, enrolled in at least one class at the participating high schools, and present on the day of data collection. Exclusion criteria included a previous diagnosis of diabetes, non-English speaking, or being unable to provide consent as determined by the RN on initial contact. Only one participant was excluded due to a previous Type 1 diabetes diagnosis.

**Table 1:** Response Rates

School	Number of Students Eligible	Number of Student consented	Percentage
Site 1	280	59	21
Site 2	87	52	60
Site 3	106	49	46
Site 4	241	94	39
Site 5	521	172	33

### Anthropometry, blood pressure, and diabetes measurements

Anthropometric measurements including weight and height were assessed and used to calculate body mass index (BMI) [16]. Following World Health Organization [17] guidelines, BMI was interpreted as standard deviations and percentiles in relation to age and sex. Those with a BMI greater than 1 standard deviation for their age were considered overweight and those with a BMI greater than 2 standard deviations for their age were considered obese.

Blood pressure was measured manually by an RN using a standard clinical sphygmomanometer. For adolescents ≤17 years, hypertension was defined as a mean systolic blood pressure (SBP) and/or diastolic blood pressure (DBP) at or above the 95th percentile for sex, age, and height [18]. Those with an SBP or DBP measured at or greater than the 90th percentile but less than the 95th percentile were considered to have prehypertension. For those who were ≥ 18 years old, abnormal increases in BP were defined as follows: mean SBP ≥ 140 mmHg or DBP ≥ 90 mmHg was high/hypertensive and mean SBP of 130–139 mmHg and/or a DBP of 85–89 mmHg was prehypertensive [19].

A validated cobas hemoglobin A1c (HbA1c) point of care test assay test was used to assess blood glucose levels [20]. Diabetes Canada criteria were used to classify adolescents into normal, high risk, prediabetes, and type 2 diabetes categories [21]. HbA1c levels < 5.5% (36 mmol/mol) were considered normal, 5.5–5.9% (37–41 mmol/mol) were considered high risk for diabetes, 6.0–6.4% (42–46 mmol/mol) were considered prediabetic, and above 6.5% (48 mmol/mol) were classified as type 2 diabetic [21, 22]. Any participant who presented with an HbA1c level ≥ 5.5% (37 mmol/mol) was referred to a physician for further investigation and other recommended tests to diagnose prediabetes and type 2 diabetes.

Other risks factors for the development of type 2 diabetes included in the screening were demographic data and a history of diabetes in a first- or second-degree relative. The adolescents were asked about their age, gender with which they identify, ethnicity, and personal medical history of diabetes.

The current literature suffers from significant disparities regarding the terms used to define ethnic groups. This study defines ethnicity on the basis of cultural characteristics, such as shared language, ancestry, religious traditions, dietary preferences, and history [23]. Those who self-identify as Indigenous include First Nations (North American Indian), Métis, or Inuit peoples, and/or those who registered under the Indian Act of Canada, and/or those who report membership in a First Nation or Indian band [24]. The term European defines adolescents who are of European descent, and are non-minority and/or Caucasian; and the term Filipino defines those who were born in and/or immigrated from the Philippines.

### Analysis

Descriptive and inferential statistics were computed using the Statistical Package for Social Sciences (SPSS v.22.0, IBM, New York, USA) to establish risk and prevalence for prediabetes and type 2 diabetes in the adolescent sample. Additionally, chi-square analyses were conducted to investigate if the risk factors of hypertension, obesity, and family history occurred at higher frequencies in certain ethnicities. Comparisons were not reported with the African, and Asian, and “other” (defined below) categories due to small group sizes. Pairwise comparisons were also conducted along with Bonferroni corrections. Significant chi-square comparisons between the three groups (European, Indigenous, and Filipino) were identified. For each significant pairing, an odds ratio and confidence interval were calculated.

## Results

A total of 396 adolescent participants were screened for prediabetes and type 2 diabetes. The sample had a similar representation of male (*N* = 179) and female (*N* = 217) participants ranging in age from 14 to 19 years (mean = 16.65). Demographic data, family history, and ethnicity are reported in Table 2.

**Table 2:** Demographics

Gender	Male	179	45.2
	Female	217	54.8
Ethnicity	Indigenous	163	41.2
	Filipino	91	23.0
	Asian	20	5.1
	African	22	5.6
	European	78	19.7
	Middle Eastern	6	1.5
	Central American	2	0.5
	South American	1	0.3
	Mixed Ethnicity	13	3.1
Age	14	27	6.8
	15	51	12.9
	16	77	19.4
	17	135	34.1
	18	94	23.7
	19	12	3.0
Family History of Diabetes and Prediabetes	Yes	108	27.3
	No	288	72.7

Many participants self-identified as Canadian as their families had been in living in Canada for many years. However, when asked more about their family traditions and heritage, they were able to specify their ethnicity. Some adolescents described their ethnicity as mixed or having more than one family ethnicity and heritage. Most adolescents self-identified as Indigenous (41.2%), Filipino (23%), European (19.7%), African (5.6%), or Asian (5.1%). An “other” category was created to capture the small numbers of adolescents (total 5.4%) in other ethnic groups, which included Latino and Middle Eastern participants as well as those who self-identified with more than one ethnicity. The percentage of each ethnicity is reported in Table 1.

### HbA1c measurements

**Fig. 1:**
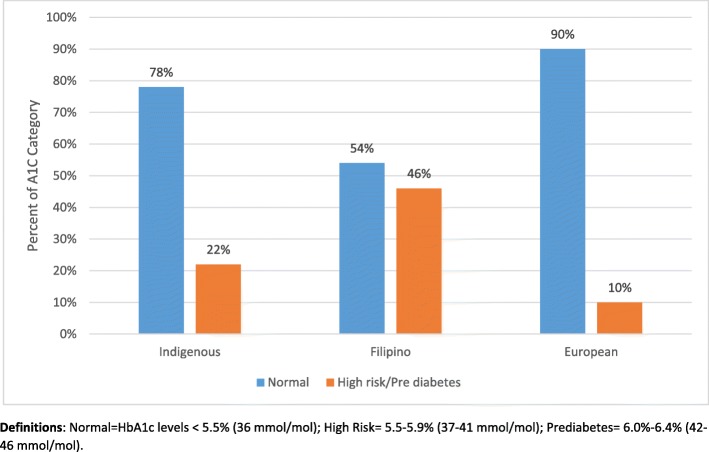
High Risk/Prediabetes by Ethnic Group

HbA1c levels ranged from 4.0 to 6.4 (mean 5.27, SD ± 0.35). For the overall sample, 72.8% (*n* = 289) of adolescents were categorized as having normal HbA1c levels, 27.3% (*n* = 89) had increased HbA1c levels and were considered high risk (5.5–5.9%), and 2.3% (*n* = 10) were classified in the prediabetes range (6.0–6.4%). Blood glucose measurements for each ethnic group is reported in Fig. 1. In the overall comparison of the three ethnic groups (European, Filipino, and Indigenous), chi-square analyses indicated statistically significant differences in terms of ethnicity (X^2^ = 31.85, df = 3, *p* < 0.0001). Significant pairs were determined in the pairwise comparison. The odds of adolescents from the Filipino group having increased HbA1c levels were 3.13 times higher compared to the Indigenous group; the odds of adolescents from the Filipino group were 7.50 times higher than the European group. Significant odd ratios and confidence intervals are reported in Table 3. However, no significant differences were noted in terms of HbA1c levels in the comparison of the European and Indigenous groups.

**Table 3:** Odds Ratios

Blood Glucose Comparisons by Ethnicity	
Comparison	Odds Ratio (CI)
Filipino > European	7.50 (2.49–22.61)*
Filipino > Indigenous	3.13 (1.51–6.51)*
Indigenous > European	0.42 (0.14–1.23)
Blood Pressure Comparisons by Ethnicity	
Race Ethnicity	Odds Ratio (CI)
European > Indigenous	4.14 (1.95–8.78)*
Filipino > Indigenous	2.94 (1.45–5.99)*
European > Filipino	1.41 (0.63–3.14)

Abbreviations: *P < 0.001, Bonferroni corrections were applied for the pairwise comparisons

### Body mass index

**Fig. 2:**
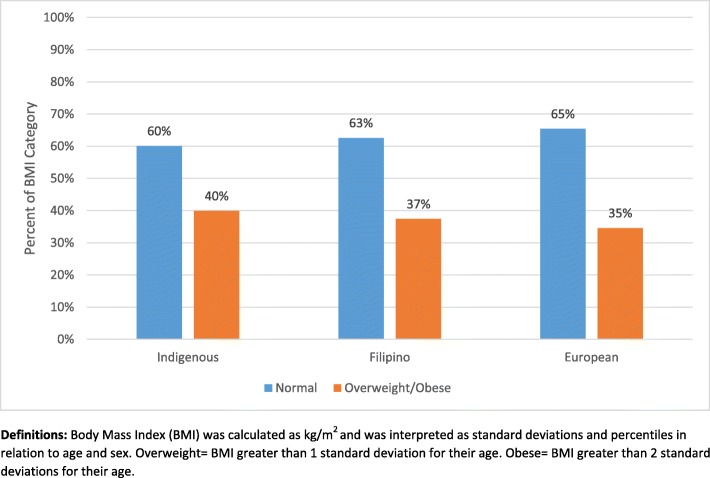
Body Mass Index By Ethnicity

BMI for the overall population ranged from 15.4 to 50.5 (mean = 24.20, SD ± 5.55). For the overall sample, 63.4% (*n* = 251) of adolescents were categorized as normal weight, 17.9% (*n* = 71) as overweight, and 18.7% (*n* = 74) as obese. The rates of overweight/obesity for each ethnic group are reported in Fig. 2. Chi-square analyses indicated no statistically significant differences between ethnicities.

### Blood pressure measurements

**Fig. 3:**
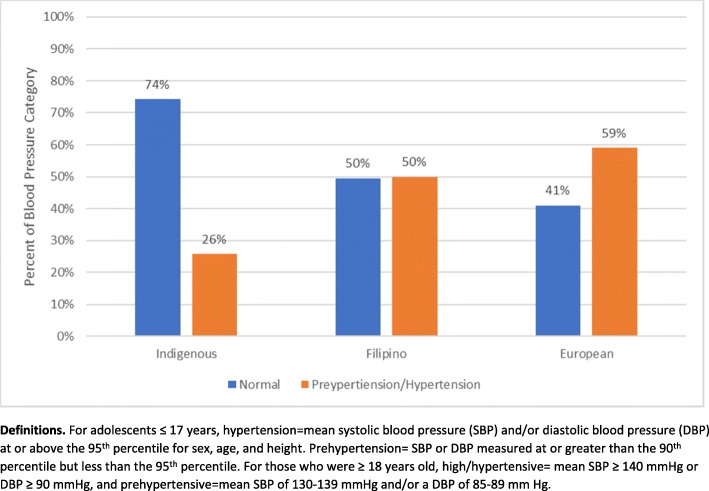
Prehypertension/Hypertension by Ethnic Group

With respect to blood pressure, more than half of the overall sample (60.6%) of adolescent participants were classified in the normal range, with notable prevalence of prehypertension (17.7%) and hypertension (21.7%). Blood pressure classification for each ethnic group is reported in Fig. 3. In the overall comparison of the three ethnic groups (European, Filipino, and Indigenous), chi-square analyses indicated statistically significant differences in terms of ethnicity (X^2^ = 30.56, df = 3, *p* < 0.0001). Significant pairs were determined using pairwise comparisons. Specifically, the odds of adolescents from the European group having prehypertension/hypertension were 4.14 times higher compared to the Indigenous group; the odds of adolescents from the Filipino group having prehypertension/hypertension were 2.94 times higher than the Indigenous group. Lastly, the European group had a higher prevalence of prehypertension/hypertension than the Filipino group but the pairwise comparison of the European and Filipino groups was not significant due in part to limited power. Significant odds ratios and confidence intervals are reported in Table 3.

### Risk factors

**Fig. 4:**
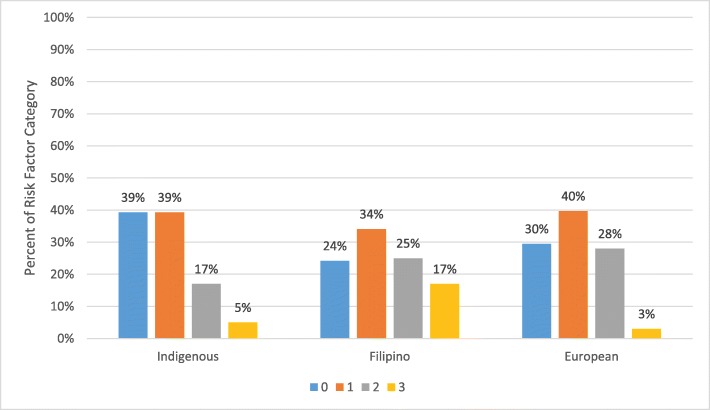
Risk Factors (overweight/obese, prehypertension/hypertension, elevated HbA1c measurements) by Ethnic Group

When ethnic differences were further analyzed, the Filipino group had the highest number of adolescents (17%) who presented with three risk factors, including overweight/obese, prehypertensive/hypertensive, and an elevated blood glucose. In addition, higher numbers of adolescents of European ancestry had at least one (40%) or two (28%) risk factors for prediabetes and type 2 diabetes. Participants with increased risk factors were categorized by ethnicity (Fig. 4).

## Discussion

This paper analyzed data from two published studies to examine the prevalence of undiagnosed prediabetes and type 2 diabetes and the associated risk factors among various ethnic adolescent groups. In the present study, several statistically significant differences were identified in relation to ethnicity. Many of the adolescents had increased HbA1c levels; the highest prevalence was among the Filipino group followed by the Indigenous and European groups. Notable prevalence of prehypertension/hypertension were reported in the overall sample with adolescents of European decent presenting with significantly higher blood pressure than the Filipino and Indigenous groups.

Research clearly illustrate that the prevalence of type 2 diabetes has increased over time among ethnic adolescents [7]. For example, an American study investigated type 2 diabetes in Pacific Island (Filipino) children ages 10–19 and found a rate of 0.46 per 1000 persons; no data were available for prediabetes [25]. Another USA population-based study estimated the prevalence of diagnosed type 2 diabetes and found that Asian (Filipino, Chinese) and Pacific Islander and Asian Pacific-Islander (*n* = 245) populations had a prevalence (existing cases) of 0.52 per 1000 youth aged 10–19 years [10]. Similar prevalence rates of type 2 diabetes were found in youth of Asian descent (12.2 per 100,000) living in England and Wales [26]. In the current report, adolescents of Filipino ancestry had a significantly higher prevalence of elevated HbA1c (46%) as compared to adolescents in the European (10%) and Indigenous (22%) groups. Early onset type 2 diabetes is associated with greater lifetime risk of diabetes-associated complications [3]. The unique findings presented in this report illustrate significantly higher prevalence of undiagnosed elevated blood glucose/prediabetes in the Filipino group than a previous Canadian study that reported incidence of 10% in the Asian ethnic group [1]. Results demonstrated in the present study confirm the need for early screening for prediabetes and the associated risk factors in adolescents of ethnic decent, with particular attention to youth of Filipino families. In addition, prevention efforts are urgently needed to halt the projected four-fold increase in the prevalence of type 2 diabetes in adolescents as well as the heightened consequences of diabetes-related mortality and morbidity at younger ages [27].

Another new and concerning finding was the prevalence of elevated blood pressure among the ethnic groups. Youth of European descent had significantly higher blood pressure as compared to adolescents of Filipino or Indigenous descent, with prevalence in both the European (59%) and Filipino (50%) groups being double those in previous reports [28]. These ethnic groups also presented with other risk factors such as overweight/obesity; specifically, many of the European (27%) and Filipino (27.5%) adolescents presented with both high blood pressure and overweight/obesity. Prevalence of hypertension can be 20–30% at initial diagnosis of type 2 diabetes [29, 30]. The current findings are concerning as hypertension may account for 35–75% of micro-macrovascular problems, including retinopathy, neuropathy, diabetic kidney disease, and myocardial infarction [29], and often clusters with other comorbidities such as obesity and hyperglycemia [3]. The high prevalence of undiagnosed hypertension illustrate the urgent need for screening to prevent the serious complications and adverse health problems associated with elevated blood pressure that will reduce quality of life for these adolescents. Children from all of these ethnic groups are at significant risk of cardiovascular disease as they enter adulthood, suggesting that a concerted effort is required to minimize or prevent elevated blood pressure in adolescents [7].

Similar to blood pressure, the prevalence of overweight and obesity among adolescents is disconcerting. In the USA, an estimated one-third of adolescents are overweight and 17% of these youth would also be categorized as obese [31, 32]. A similar prevalence of overweight/obesity (30%) was found in Canadian youth [33]. The current findings concur with previous studies and confirm the trend of overweight/obesity in ethnic youth. Specifically, the prevalence of overweight/obesity in youth of Indigenous (40%), Filipino (37%), and European (35%) descent are alarming considering the increasing prevalence of type 2 diabetes are directly coincident with global increases of overweight/obesity in ethnic children and adolescents [34, 35].

### Limitations

Although the data analyzed were from two studies with a largely multicultural sample, small numbers of adolescents self-identified as Asian, Latino, African, and mixed ethnicity. Therefore, a meaningful comparison with these ethnic groups was not possible. In addition, there may have not been sufficient power to detect significant differences with some of the ethnic comparisons. Future research with a larger sample of these particular ethnic groups is needed to explore undiagnosed prediabetes and the associated risk factors. Although some Indigenous adolescents lived in the urban centre, the majority of those represented in the data analyzed in this article lived in rural and remote communities and have different living circumstances that could influence the results. Therefore, future research should explore possible differences between urban versus rural living, particularly for adolescents of Indigenous descent. Data collection took place in the schools using the cobas hemoglobin A1c (HbA1c) point of care test assay test, therefore, only using this measure of blood glucose is a limitation of these results. However, Diabetes Canada (2018) reported that the HbA1C ≥ 6.0% is able to identify children with type 2 diabetes at 86% sensitivity and 85% specificity and had similar screening efficacy to Fasting Plasma Glucose, when compared to the gold standard 2-h Oral Glucose Tolerance Test (p. S248). It is recommended that the HbA1C be used in combination with other tests and for this reason all the adolescents who presented with high measures of blood glucose were referred for further investigation.

## Conclusion

The prevalence of type 2 diabetes in youth is increasing worldwide, with higher prevalence among adolescents of ethnic groups. The trend of prediabetes among specific ethnic groups is also increasing, with many adolescents remaining undiagnosed. The findings reported herein indicate significant numbers of Filipino youth with undiagnosed elevated blood glucose levels. The elevated blood pressures in the European and Filipino groups were also significantly higher than previously reported prevalence. As this is the first report of undiagnosed prediabetes and elevated blood pressure in youth of these ethnic groups in Western Canada, the results confirm the need for effective interventions to prevent the future health burden among these adolescent ethnic groups. Additional research is warranted to investigate the unique contributions of genetic and/or environmental factors among each ethnic group to early onset hypertension and prediabetes/type 2 diabetes.

## Data Availability

The datasets generated and/or analyzed during the study are not publicly available due to confidentiality but are available from the corresponding author on reasonable request.

## Abbreviations

BMI: Body Mass Index
BP: Blood Pressure
DBP: Diastolic Blood Pressure
HbA1C: Hemoglobin A1C
NHB: Non-Hispanic Black
PI: Principal Investigator
RN: Registered Nurse
SBP: Systolic Blood Pressure
USA: United States of America
